# Profiling of Serum and Urinary MicroRNAs in Children with Atopic Dermatitis

**DOI:** 10.1371/journal.pone.0115448

**Published:** 2014-12-22

**Authors:** Yani Lv, Ruiqun Qi, Jing Xu, Zhenghong Di, Heng Zheng, Wei Huo, Li Zhang, Hongduo Chen, Xinghua Gao

**Affiliations:** 1 Department of Dermatology, No. 1 Hospital of China Medical University, Shenyang, Liaoning, China; 2 Department of Dermatology, Shengjing Hospital of China Medical University, Shenyang, Liaoning, China; CNRS-University of Toulouse, France

## Abstract

**Background:**

Atopic dermatitis (AD) is the most prevalent chronic inflammatory skin disease in children characterized by dermatitis and pruritus. MicroRNAs (miRNAs) have been shown as great potential biomarkers for disease fingerprints to predict prognostics. We aimed to identify miRNA signature from serum and urine for the prognosis of AD patient by genome-wide miRNA profiling analysis.

**Methods:**

Serum and urine from 30 children with AD and 28 healthy children were collected and their genome-wide miRNA expression profiles were measured by TaqMan-based array and confirmed by quantitative real-time PCR. Inflammatory factors in serum were detected by Antibody Array System.

**Results:**

miR-203 and miR-483-5p were significantly up-regulated in serum of children with AD compared with healthy children. The level of miR-483-5p in serum was significantly associated with other atopic conditions, such as rhinitis and/or asthma. However, miR-203 was markedly decreased in urine of children with AD compared with healthy children. Down-regulated miR-203 in urine was significant associated with abnormal level of serum IgE in AD patients. 7 inflammatory factors in serum were altered in children with AD compared with healthy children. Up-regulated miR-203 in serum was significantly associated with increased sTNFRI and sTNFRII.

**Conclusions:**

Up-regulated miR-483-5p in serum may be indicative of other atopic conditions in children with AD. Down-regulated miR-203 in urine may serve as a biomarker for the severity of inflammation in children with AD.

## Introduction

Atopic dermatitis (AD) is a chronic and recurrent inflammatory skin disease characterized by typical distribution of skin lesions and severe pruritus. AD may occur as an independent entity or as a part of a triad of conditions including asthma, allergic rhinitis (hay fever), and a chronic dermatitis (eczema). AD may start at any ages but is more common in infants and young children, about 50% to 90% of AD occurs by the age of 6 to 12 months [1]. And about half of AD infants develop into allergic respiratory condition by the age of five. In clinic, severity scoring of atopic dermatitis (SCORAD) score is usually used to assess severity of this disease, and it is comprised of three major factors: extent of involvement, intensity of lesions and subjective signs [2]. Recently, it was reported that serum IgE level and peripheral eosinophilic granulocyte are remarkably increased in most of AD patients, and they can serve as reference laboratory parameters [3].

MicroRNAs (miRNAs) are a class of small, endogenous 22–25 nt RNA molecules that bind to specific mRNAs to inhibit translation and promote mRNA degradation. miRNAs function in post-transcriptional regulation in RNA-induced silencing complex (RISC) and control physiological and pathological processes in various diseases [4]. The expression profile of miRNAs can be organ or cell specific. For example, most miR-203 is exclusively expressed in keratinocytes, while most miR-146 expressed in immune cells [5]. Aberrant miRNAs expression has been found in many cancers [6] and immunologic and inflammatory disorders, such as psoriasis, lupus erythematosus, and airway inflammation [5], [7], [8].

Recently, a panel of miRNAs was identified to over-express in infiltrating cells of lesional skin in AD patients. For example, miR-155 was over-expressed in lesion of AD, and it increased the proliferative response of T cells through down-regulating of cytotoxic T lymphocyte-associated antigen 4 (CTLA-4) expressions [9]. Blockade of miR-126 was found to suppress Th2-mediated allergic airway inflammation through inhibition of IL-4, a Th2 cytokine, thus suppressing development of asthma [8]. These findings demonstrate that miRNAs may take regulatory roles in pathogenesis of the allergic diseases albeit through different pathways. An enriched miRNAs fraction has been shown stably in serum and the miRNAs expression is concordant with blood cells under normal conditions. Moreover, miRNAs are found in other body fluids as well, including urine, tear, and amniotic fluid [10]. These remarkable characteristics of miRNAs have made miRNAs as promising biomarkers for various diseases [11].

In the present study, we performed a genome-wide miRNAs profiling in serum and urine from children with AD and screened out some potential biomarkers. Up-regulated and down-regulated miRNAs in serum and urine were not concomitant with those from the lesional skin as previously reported [9]. We found that some miRNAs in serum, such as miR-203 and miR-483-5p, were significantly up-regulated in children with AD compared to healthy children. Paradoxically miRNA-203 was significantly down-regulated in urine from AD patients. In addition, in order to find out the link between miRNAs expression and immune response, we also detected the global inflammatory factors in serum and our results showed that ur-regulated miR-203 in serum was significantly associated with the expression of sTNFRI and sTNFRII.

## Materials and Methods

### Patients and controls

30 children with AD were enrolled according to diagnostic criteria defined by Williams. 28 healthy children were enrolled as control. Healthy children were carefully chosen from patients undergoing pediatric surgery, excluding those with history of atopic disorders or inflammatory skin diseases and those with their total IgE levels in serum above the normal limit (IgE: 1.31–165.3 IU/ml). There were 24 boys and 4 girls (aged 6 months to 6 years, mean 2.1±0.16 years) in control group. Both patients and controls were of Chinese Han origin. Severity of childhood AD was assessed with Scoring Atopic Dermatitis SCORAD index. AD patients were classified into four subtypes according to the age of onset, level of IgE and eosinophil count in peripheral blood, and other concurrent allergic diseases in patients or their family members. All subjects had not been received systemic corticosteroids or other immuno-suppressants for the last 3 months. The study was approved by the China Medical University Ethics Committee, and informed written consent was obtained from their parents.

### Sample collection and handling

Peripheral venous blood and urine samples were derived from patients at No. 1 Hospital of China Medical University and ShengJing Hospital of China Medical University. Blood and urine samples were obtained and centrifuged at 2500 g for 10 min within 2 hours after collection. Serum and cellar fraction, urinary supernatant and urinary sediment were separated and stored at −80°C for further use.

### Taqman-based array and real-time quantitative PCR

Total RNA was extracted from serum and urine samples with miRNeasy kit (#217004, QIAGEN) according to the manufacturer's protocol for liquid samples. In general, the yield was 2–10 ng/ul. The global miRNA profiling was performed by using the TaqMan Low Density Array (TLDA) Human microRNA Panel version 1.0 (Capitalbio, Beijing, China). Quantification of miRNAs by means of TaqMan Real-Time PCR was carried out as described by the manufacturer (Capitalbio, Beijing, China). The microRNA profiling array data was accessible by GEO: #GSE62406.

### Global normalization

Raw cycle threshold (Ct) values were calculated using SDS 2.3 and RQ manager 1.2 software (Applied Biosystems) and applied automatic baselines and threshold settings. The Ct value greater or equal to 35.0 was cutoff. To perform global normalization, all the Ct values after cutoff were imported into StatMiner 4.2 (Integromics Inc., Philadelphia, PA). miRNAs detected in serum and urine samples were used for global normalization. Global normalization process calculates the mean Ct value from fully measurement of miRNAs in each sample and subtracts this value from the Ct value of each individual miRNA from the same sample. The resulting value is the ΔCt. The ΔΔCt was then calculated by subtracting average ΔCt of the normal controls from ΔCt of children with AD.

### Inflammatory factors antibody array system

Serum samples were analyzed by an antibody array system (Ray Bio Human Inflammatory Antibody Array III Kit, Ray Biotech, USA) according to the manufacturer's instructions. Briefly, the array membranes were blocked with 1 x blocking buffer for 30 min and then incubated overnight with 1 ml sample at 4. After incubation, the membranes were washed three times with 2 ml 1 x Wash Buffer I followed by two washes with 2 ml 1 x Wash Buffer II at room temperature with shaking. The membranes were then incubated with 2 ml 1∶500 diluted biotin-conjugated antibodies for 2 h at room temperature and washed as described above; then followed by incubation with 1 ml 1∶40,000 diluted streptavidin-conjugated peroxidase for 1 h at room temperature. After a thorough wash, the membranes were exposed to a peroxidase substrate for 5 min in the dark prior to imaging. The membranes were exposed to an X-ray film within 30 min of exposure to the substrate. Signal intensities were quantified with Gel-Pro analyzer software.

### Luciferase activity assay

The assay was performed as previous described [12]. In brief, 10^4^ cells were transfected with lipofectmine 2000 (Invitrogen, Carlsbad, USA) according to the manufacturer's instruction. 200 ng of firefly luciferase reporter plasmid DNA, 10 uM precursor miR-203 oligos and 0.6 ng of Renilla luciferase reporter plasmid pRL-TK were transfected into 24-well dish. Cells were incubated for 24 h, and luciferase activity was measured with Dual Luciferase Reporter Assay kit (Promega) according to the manufacturer's instructions. The firefly luciferase activity values were normalized to the Renilla luciferase activity values that reflect transfection efficiency. Data are presented as mean values (± SD).

### Statistical analysis and ROC curve

The ΔCt was calculated and a heat map analysis was performed with complete-linkage hierarchical clustering using StatMiner 4.2 (Integromics Inc., Philadelphia, PA). A non-Parametric Wilcoxon test was used to compare difference in serum or urine miRNAs between children with AD and control individuals. A false discovery rate (FDR) was adjusted with the Benjamini-Hochberg method. An adjusted two tailed *P*-value<0.01 was considered significant. Scatter plots were obtained by using GraphPad Prism 5.01 software (GraphPad Software, Inc., La Jolla, CA). Receiver operating characteristic (ROC) curves and the area under the ROC curve (AUC) were used to assess the ability of serum or urine miRNA levels for detecting. ROC analysis was performed by the SPSS 18.0 (SPSS Inc., Chicago, IL). Differences among the groups were performed by one-way ANOVA test. Correlation analysis was performed to assess the correlation between miRNAs expression levels and clinical parameters in children with AD. Differences were deemed statistically significant at P<0.05.

## Results

### General information of enrolled children with AD

As shown in Table 1, 30 children with AD were enrolled in this study. 22 were boys and 8 were girls, ranging from 6 months to 6 years (mean 1.9±0.32 years). 21 in 30 had disease onset within two months after birth, while the rest of 9 had onset age of over two months. 16 in 30 had abnormally high level of IgE in serum, and 21 in 30 had abnormally high eosinophil count in peripheral blood. 9 patients had concurrent allergic rhinitis and one had asthma. 20 children had no other symptoms but all of their family members had atopic conditions.

**Table 1:** Demographics of childhood AD.

No. of AD	Gender (F/M)	Age (months)	Onset age (months)	Serum IgE (IU/mL)	Eosinophilic Count (.04–.49×10^9^/L)	SCORAD Index	Concurrent with other atopy	Familial atopy
1	M	8.2	≤1	37.78	0.15	36.6	No	Yes
2	M	6	≤1	159.13	2.18	31.7	No	Yes
3	M	8	≤2	1963.90	18.90	21.1	Yes	Yes
4	M	60	≤1	22.37	0.29	40.6	Yes	No
5	M	6	≤2	1567.80	2.34	31.9	No	Yes
6	M	6	≤1	64.76	0.65	32.3	Yes	Yes
7	M	69	>2	2.80	0.05	21.6	No	Yes
8	M	30	≤1	730.10	0.39	20.0	Yes	Yes
9	M	10	≤1	1225.31	9.58	29.3	No	Yes
10	F	7.5	>2	35.16	0.16	28.6	Yes	No
11	F	68	≤1	998.65	2.11	31.4	No	Yes
12	F	7	≤1	1879.14	17.60	55.7	No	Yes
13	M	6	≤2	276.95	1.92	40.4	No	Yes
14	F	36	≤2	37.27	0.38	27.3	Yes	No
15	M	45	≤1	183.28	2.95	45.0	No	Yes
16	M	6	≤1	414.62	1.38	37.1	No	Yes
17	F	56	>2	106.38	0.31	27.5	Yes	Yes
18	M	19	>2	768.66	2.29	27.5	No	Yes
19	F	7	≤1	125.79	0.78	45.5	No	Yes
20	M	31	>2	45.65	0.21	44.8	No	Yes
21	F	39	≤1	87.79	0.43	24.9	No	Yes
22	F	24	≤1	1023.63	9.42	25.9	Yes	Yes
23	M	52	>2	1674.34	16.60	45.9	No	Yes
24	M	36	>2	1259.21	11.20	22.5	No	Yes
25	M	7	≤1	617.91	2.34	43.0	Yes	No
26	M	7	>2	19.52	0.62	48.6	Yes	No
27	M	10	≤1	59.07	0.87	36.8	No	Yes
28	M	24	≤1	322.81	1.24	21.8	No	Yes
29	M	7	>2	155.11	1.97	29.5	No	Yes
30	M	11	≤1	1260.04	15.30	22.9	No	Yes

Normal ranges: IgE: 1.31–165.3 IU/ml; Eosinophil: 0.04–0.49×10^9^/L.

### Disproportionate expression of miRNAs in serum and urine from children with AD

Three miRNAs, miR-374a, miR-374b and let-7d, were stable and could serve as ideal endogenous normalizers for circulating miRNAs [13]. The present study confirmed the stable expression of the above three miRNA in serum and urine from both patients and controls, and miR-374a was chosen as an internal normalizer to quantify the relative amount of miRNAs in serum and urine.

**Figure 1:**
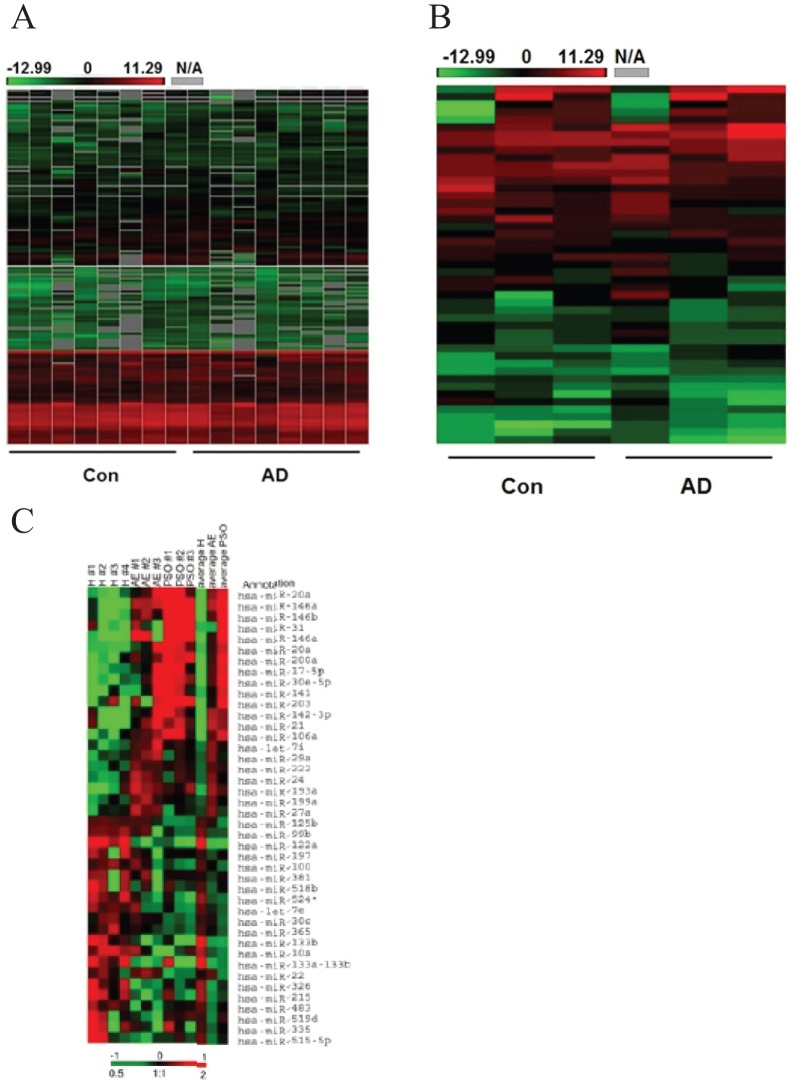
Genome-wide miRNA expression in children with AD. **A**. Heatmap analysis showing miRNAs gene expression profile in serum samples in 8 children with AD and 8 healthy children. **B**. Heatmap analysis showing miRNAs gene expression profile in urine samples in 3 children with AD and 3 healthy children. **C**. Heatmap analysis showing significantly differentially expressed miRNAs in children with AD.

miRNAs expression in serum from patients (n = 8) and controls (n = 8) and miRNAs expression in urine from patients (n = 3) and controls (n = 3) were compared respectively using miRNAs arrays. Our analysis showed that 116 miRNAs in urine and 255 miRNAs in serum were detected by the Taqman-based array (Fig. 1). 10 serum miRNAs and 17 urinary miRNAs were identified as significantly differentially expressed miRNAs between children with AD and controls (9 up-regulated and 1 down-regulated miRNAs in serum and 10 up-regulated and 7 down-regulated miRNAs in urine), as listed in Table 2. Intriguingly, miR-203, miR-483-5p and miR-205 were coexisting in both serum and urine groups. miR-483-5p and miR-205 were up-regulated in both serum and urine in children with AD compared with the controls. miR-203 was up-regulated in serum but was markedly decreased in urine of children with AD (Table 2).

**Table 2:** miRNAs differentially expressed in serum and urine of childhood AD compared to controls (P<0.05).

Serum			Urine		
miRNAs	−△△CT	Fold	miRNAs	−△△CT	Fold
**Up-regulated**			**Up-regulated**		
*miR-205	2.886	7.390	MammU6	3.375	10.375
miR-539	2.505	5.678	miR-142-3p	2.672	6.373
miR-122	2.352	5.106	miR-20a	2.231	4.695
*miR-203	2.352	5.106	miR-548c-3p	1.793	3.465
*miR-483-5p	2.262	4.798	*miR-205	1.487	2.803
miR-134	2.086	4.246	miR-19a	1.329	2.512
let-7g	1.838	3.575	*miR-483-5p	1.118	2.170
miR-495	1.420	2.676	miR-222	1.104	2.149
miR-642	1.408	2.653	miR-92a	1.103	2.148
			miR-548a-3p	1.101	2.145
**Down-regulated**			**Down-regulated**		
miR-590-5p	-0.876	0.545	*miR-203	−2.695	0.154
			miR-125a-5p	−1.942	0.260
			miR-886-3p	−1.317	0.401
			miR-184	−1.302	0.406
			miR-886-5p	−1.253	0.420
			miR-26a	−1.107	0.464
			miR-194	−1.101	0.466

* miRNAs expressed in both serum and urine.

### miR-483-5p and miR-203 were up-regulated in serum of children with AD

To validate the array profiling, we performed quantitative real-time PCR analysis of selected up-regulated expression levels of miRNAs including miR-203, miR-205, miR-483-5p, miR-134 and miR-122 in serum from control children (n = 28) and childhood AD (n = 30). Previously these miRNAs had been reported to be highly expressed in AD lesional skin [5], and involved in immuno-regulatory network [14]–[17].

**Figure 2:**
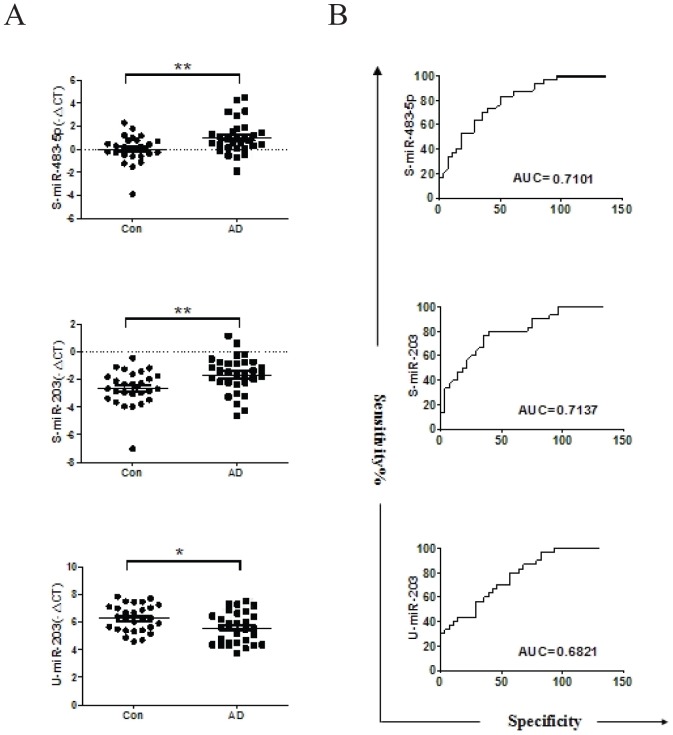
Validation of selected miRNA expression levels. **A**. Expression of two miRNAs in serum (S-miR-483-5p and S-miR-203) and one in urine (U-miR-203) were measured by quantitative real-time PCR were in 30 children with AD and 28 healthy children. The 2^-△△CT^ method in one subject with children with AD was normalized to 1. miR-374a expression was considered as control. *P<0.05, **P<0.01. **B**. ROC and AUC analysis for the ability of serum miRNAs (S-miR-483-5p and S-miR-203) and urine miRNA (U-miR-203) were performed. ROC Curve analysis shows the sensitivity (y-axis) and specificity (x-axis) of a standard of potential biomarkers.

**Figure 3:**
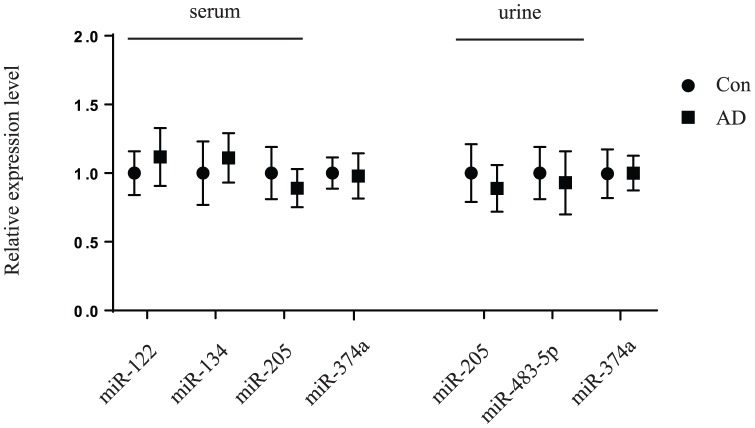
Validation of other miRNA expression levels by quantitative real-time PCR. Selected miRNAs (miR-122, miR-134, miR-205 in serum and miR-205, miR-483-5p in urine) were measured in 30 children with AD and 28 healthy children. The 2^−△△CT^ method in one subject with children with AD was normalized to 1. miR-374a expression levels in serum and in urine were considered as control. Data are presented as mean values (± SD).

**Figure 4:**
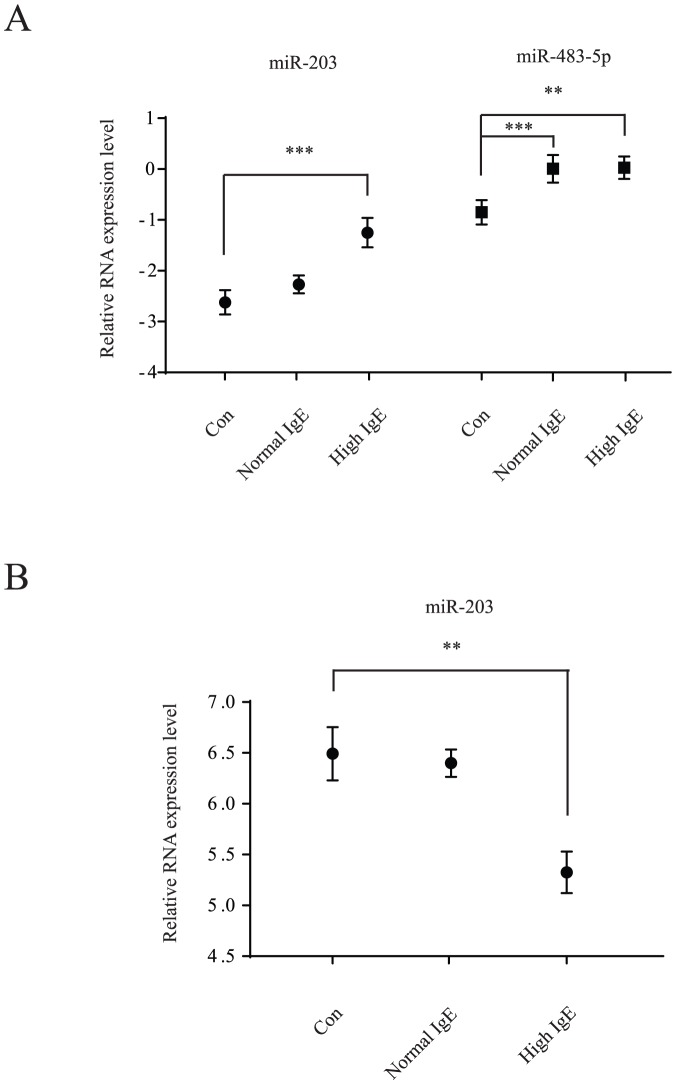
Groupwise comparison between IgE level and miRNA expression level in children with AD. **A**. miR-203 and miR-485-5p expression level in serum were measured in patients with higher IgE level and normal IgE level by quantitative real-time PCR. Data are presented as mean values (± SD). **P<0.01, ***P<0.001. **B**. miR-203 expression level in urine was measured in patients with higher IgE level and normal IgE level by quantitative real-time PCR. Data are presented as mean values (± SD). **P<0.01.

Consistently, miR-483-5p and miR-203 were significantly up-regulated (p<0.05) in serum from children with AD compared with the controls (Fig. 2A). These two miRNAs were further analyzed by the receiver operating characteristic curve (ROC). The values of area under the receiver operating characteristic curve (AUC) for these miRNAs were 0.7101 and 0.7137, respectively (Fig. 2B). However miR-122, miR-134 and miR-205 did not show dramatically changed in patient samples compared to the controls (Fig. 3). Patients with higher level of IgE in serum had significant higher expression of miR-203 in serum compared to the controls (p = 0.0011), however patients with normal level of IgE had no significant difference compared to the controls (p>0.05) (Fig. 4A). Patients with higher IgE level or normal IgE level had significant higher miR-483-5p expression in serum compared to the controls (p = 0.0157, p = 0.0094, respectively) (Fig. 4A). Furthermore, in other subdivision comparison according to different confounding factors, there was no significant difference between expression levels of miR-483-5p and miR-203 and references to ages, gender, SCORAD, and the number of eosinophils (all P>0.05) (data not shown). However, up-regulated miR-483-5p in serum was significantly associated with other concurrent atopic conditions in children with AD, such as rhinitis and/or asthma, (n = 10) compared with the remaining patients (n = 20) (relative amount -0.1887 vs -1.3367, P<0.05) (Table 3).

**Table 3:** Correlation analysis between childhood AD with atopic disease and childhood AD alone (T-test).

	Onset age			Combine with Atopic disease		
Inflammatory Factors	(≤2 m)	(>2m)	P value	Personal history of atopic disease	Family history of atopic disease	P value
**miR-483**	−1.092	−1.016	0.902	−0.189	−1.337	**0.044**
**miR-203**	−1.750	−1.556	0.717	−2.120	−1.484	0.242
**urine-miR-203**	5.597	5.117	0.986	5.843	5.661	0.321

### miR-203 is down-regulated in urine of children with AD

We also performed quantitative real-time PCR analysis on the selected miRNAs from urine (miR-203, miR-205 and miR-483-5p). Our result showed that only miR-203 was significantly down-regulated (p<0.05) in urine from children with AD compared with the controls (Fig. 2A). ROC analysis was performed and the AUC for this miRNA was 0.6821(Fig. 2B). However, miR-205 and miR-483-5p in urine did not shown any significant changes between these two groups (Fig. 3). Patients with higher level of IgE in serum had significant lower expression of miR-203 in urine compared to the controls (p = 0.0027), however patients with normal level of IgE had no significant difference compared to the controls (p>0.05) (Fig. 4B). In other different confounding factors comparison, the expression level of miR-203 in urine from children with AD was not associated with age, gender, SCORAD, number of eosinophils, and other concurrent atopic conditions (all P>0.05) (data not shown).

### miR-203 was associated with the altered inflammatory factors in serum

**Figure 5:**
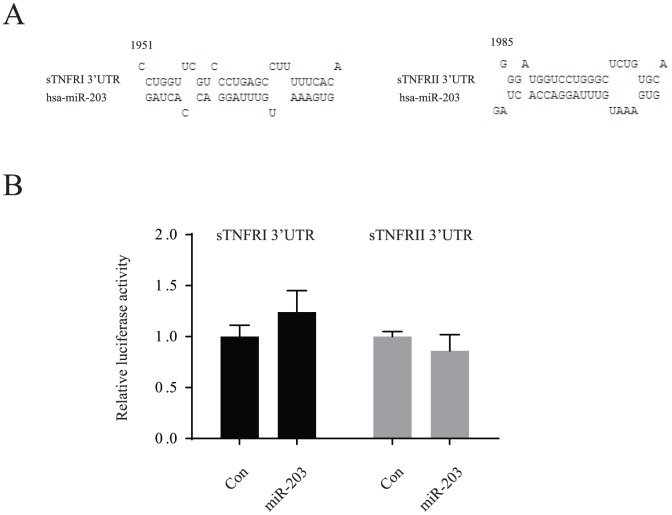
Putative targets by miR-203. **A**. Predicted miRNA sequences and their putative recognition sites within 3′ UTR of sTNFRI (NM_001065) and sTNFRII (NM_001065). **B**. Luciferase activities of sTNFRI and sTNFRII mRNA 3′UTR were not affected by miR-203. 293T cells were transfected with pGL3 vectors containing sTNFRI and sTNFRII, and Renilla luciferase expression vector, and followed the treatment of precursor miR-203 oligos and control oligos. Firefly luciferase activity was measured and normalized to the activity of Renilla luciferase.

Further, human inflammatory antibody array was performed in serum of children with AD and the controls. 7 inflammatory factors were remarkably altered in children with AD, including MIP-1b, sTNFRI, ICAM-1, IL-6sR, TIMP-2, sTNFRII and MCP-1 (Table 4). Pearson's correlation analysis between 7 Factors and 2 miRNAs in children with AD showed that miR-203 was significantly associated with sTNFRI and sTNFRII (data not shown), indicating the potential regulation role of miR-203 in regulating these two inflammatory factors. Thus, we performed the 3′UTR luciferase assay to check whether miR-203 could target the 3′UTRs of sTNFRI and sTNFRII. However, our results showed that neither sTNFRI nor sTNFRII were the direct downstream targets of miR-203 (Fig. 5). Over-expressed miR-203 did not change their luciferase activities. It indicates that there may be other mechanism linking expression of miR-203 and expression of sTNFRI/II.

**Table 4:** Correlation analysis between Clinical parameters and 7 Factors in childhood AD.

	SCORAD	
Inflammatory Factors	correlation coefficients	P Value
**MIP-1b**	0.144	0.473
**s TNF RI**	0.327	0.096
**ICAM-1**	0.087	0.668
**IL-6sR**	0.326	0.097
**TIMP-2**	0.417	**0.031**
**s TNF RII**	0.410	**0.034**
**MCP-1**	0.388	**0.045**

## Discussion

Previous studies showed that most of tissue miRNAs could be detected in body fluid. Circulating miRNAs in serum has been postulated as reliable biomarkers for disease prediction, diagnosis and severity assessment. Under normal conditions, miRNAs are released mostly from circulating blood cells in serum, while under diseased conditions, miRNAs expression profiles are different and depend on the types and natures of the diseases [10].

In the present study, only two of screened miRNAs, miR-203 and miR-483-5p, were confirmed to be statistically up-regulated in serum of children with AD. miR-203 was previously reported to be keratinocyte specific, and highly expressed in lesions of psoriasis, but not in AD [5], [9]. This paradoxical finding may be explained as follows: 1, children with AD may have different miRNAs expression profile compared to their adult counterparts; 2, over-synthesized miR-203 may over-flow in inflamed keratinocytes and be kept at constant amount in keratinocytes. Our data showed a higher serum miR-203 expression in EAD than in IAD, which suggested that alteration of miR-203 in serum may be associated with a risk of children with AD and, more possibly, and EAD.

Although the origin of miR-483-5p remains to be unexploited, previous study reported that the level of miR-483-5p in serum could be used for predicting the severity of sepsis [18]. In the present study, we found that both IAD and EAD had a higher miR-483-5p level in serum than the controls. Thus, we postulate that the level of miR-483-5p and the level of IgE in serum are relatively independent markers of AD. Furthermore, other concurrent atopic conditions had been shown to be significantly associated with up-regulated miR-483-5p in children with AD, suggesting that miR-483-5p may reflect the multi-organ/tissue involvement of the atopic conditions.

A number of studies have shown that imbalanced production of certain miRNAs is present in urine in various diseases. The detection and quantification of specific urinary miRNAs may represent a novel non-invasive tool to monitor pathological condition, such as nephrotic tumors or renal injury [19]-[21]. Contrary to increased level of miR-203 in serum of children with AD, we found a significant down-regulated miR-203 in urine, which was also negatively correlated with higher level of IgE in serum and the severity of skin inflammation. The reason for this contrary phenotype may be expression of miR-203 from other organs. Besides skin, miR-203 was highly expressed in oesophagus, and may play a role in the formation of squamous epithelia. miR-203 in mouse was preferentially expressed in the epidermis and may show its specificity in the interfollicular epidermis [5]. Furthermore, a lower level of miR-203 was detected in urine of EAD than that in IAD, which suggested that alteration of miR-203 in urine is associated with a risk of childhood AD and, more possibly, and EAD.

Excretion and origination of urinary miRNAs were still a mystery. In response to various pathophysiological stimulations, cells can actively package miRNA into micro-vesicles and release them into circulation. The high alteration of urinary miRNAs suggests that they may be primarily encapsulated in cell-secreted micro-vesicles [22], [23]. Considering recent reports describing transport of miRNAs from cell-to-cell [24] and from paracrine signaling in cells [25], it implies that synthesis, secretion and release of miRNAs between tissue/cells and circulation are interactive and complicated, and these miRNA molecules in the circulation may suggest a functional role in pathophysiologic conditions. Our result showed the up-regulated miR-203 in serum, but down-regulated miR-203 in urine of patients with AD. However, there was no any association of the level of miR-203 in serum and that in urine. Therefore, we conjectured that miRNAs may play their own roles independent relatively in serum and urine. Our previous study shown that urinary aquaporin-2 was elevated in infant AD, and was significantly associated with urinary AQP-2 level and skin dryness in infant AD [26]. Other group indicated that long-lasting AD may cause escape from the ADH–AQP-2 pathway [27]. Albeit the mechanism of down-regulated miR-203 in urine of children with AD remained unclear, urinary level of miR-203 may be used as an auxiliary biomarker to reflect the severity of inflammation of childhood AD.

In summary, we provide the first comprehensive analyses of serum and urine miRNA expression profiles in children with AD. We identified distinct miRNA expressions in serum and urine that was different from the lesional skin as previously reported [9]. Further, we identified 7 inflammatory factors in serum were altered in children with AD compared to the control, and found up-regulated miR-203 in serum was significantly associated with the level of sTNFRI and sTNFRII. This finding suggests that up-regulated miR-483-5p in serum may be indicative of other atopic conditions in children with AD, while down-regulated miR-203 in urine may serve as a biomarker for the severity of inflammation of children with AD.
