# Correction: Profiling of Serum and Urinary MicroRNAs in Children with Atopic Dermatitis

**DOI:** 10.1371/journal.pone.0119353

**Published:** 2015-03-27

**Authors:** The PLOS ONE Staff

Figure 1C is incorrect. The authors have provided a corrected version here.

**Fig 1:**
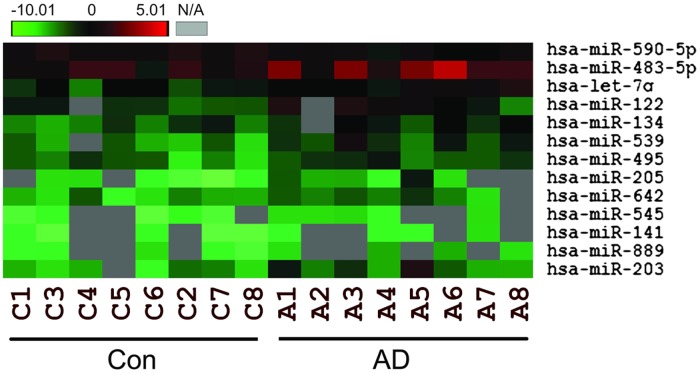
Genome-wide miRNA expression in children with AD. A. Heatmap analysis showing miRNAs gene expression profile in serum samples in 8 children with AD and 8 healthy children. B. Heatmap analysis showing miRNAs gene expression profile in urine samples in 3 children with AD and 3 healthy children. C. Heatmap analysis showing significantly differentially expressed miRNAs in children with AD.
